# Kinetics of Crystallization and Thermal Degradation of an Isotactic Polypropylene Matrix Reinforced with Graphene/Glass-Fiber Filler

**DOI:** 10.3390/molecules24101984

**Published:** 2019-05-23

**Authors:** Evangelia Tarani, George Z. Papageorgiou, Dimitrios N. Bikiaris, Konstantinos Chrissafis

**Affiliations:** 1 X-ray, Optical Characterization and Thermal Analysis Laboratory, Physics Department, Aristotle University of Thessaloniki, GR541 24 Thessaloniki, Greece; etarani@physics.auth.gr; 2 Chemistry Department, University of Ioannina, P.O. Box 1186, 45110 Ioannina, Greece; gzpap@uoi.gr; 3 Laboratory of Polymer Chemistry and Technology, Department of Chemistry, Aristotle University of Thessaloniki, GR541 24 Thessaloniki, Greece; dbic@chem.auth.gr

**Keywords:** polypropylene, graphene nanoplatelets, glass fibers, crystallization, kinetics, activation energy

## Abstract

Polypropylene composites reinforced with a filler mixture of graphene nanoplatelet-glass fiber were prepared by melt mixing, while conventional composites containing graphene nanoplatelet and glass fiber were prepared for comparative reasons. An extensive study of thermally stimulated processes such as crystallization, nucleation, and kinetics was carried out using Differential Scanning Calorimetry and Thermogravimetric Analysis. Moreover, effective activation energy and kinetic parameters of the thermal decomposition process were determined by applying Friedman’s isoconversional differential method and multivariate non-linear regression method. It was found that the graphene nanoplatelets act positively towards the increase in crystallization rate and nucleation phenomena under isothermal conditions due to their large surface area, inherent nucleation activity, and high filler content. Concerning the thermal degradation kinetics of polypropylene graphene nanoplatelets/glass fibers composites, a change in the decomposition mechanism of the matrix was found due to the presence of graphene nanoplatelets. The effect of graphene nanoplatelets dominates that of the glass fibers, leading to an overall improvement in performance.

## 1. Introduction

The continuous demand for high-performing materials in the 20th century has led to the development of polymer composites. Polymer composites usually exhibit advanced mechanical, thermal and electrical performance as a result of the reinforcing nature of the fillers [1]. Polypropylene (PP) is one of the most used semicrystalline polymers. It can be found in various applications, such as heating, piping, sanitary, film and rigid packaging, due to its low cost and good physicochemical properties. Various fillers have already been incorporated in an isotactic PP matrix in an attempt to further improve the properties of PP, and most of those works report a significant enhancement in the final properties of the composite materials [2,3,4,5].

Glass fibers (GF) are one of these fillers since they can quite efficiently reinforce most polymeric matrices due to their high stiffness and strength, their outstanding fatigue performance, and low cost [6,7,8]. For this reason, GF-reinforced composites are already a commodity in the aerospace, automotive, construction, and sporting industries. To achieve the desired properties, a GF filler content of 30–50 wt.% is used. However, the high GF loadings lead to an undesirable increase in density, decreased melt flow and increased brittleness. Carbon-based materials such as carbon fibers (CF), carbon nanotubes (CNT), carbon black (CB), graphene nanoplatelets (GNP), graphene oxide (GO) and graphene have shown excellent potential as reinforcements due to their thermal and electrical conductivity, high mechanical strength and optical properties. For this reason, even a small concentration of the above-mentioned fillers can achieve significant enhancement in the properties of the initial material. GNPs display similar mechanical, electrical, barrier and thermal properties to a few layers of graphene but are significantly cheaper to produce. The GNPs that were used in the current work are produced through an intercalation procedure from graphite flakes, which most often leads to the formation of stacks of a few layers of graphite, and has already been incorporated with success in many polymeric matrices [9,10,11,12]. 

One strategy to further enhance the properties of the PP composites, or counterbalance some of the above-mentioned disadvantages of GF fillers, is the use of a filler mixture where two or more reinforcements are used in combination [13,14,15,16]. Pedrazzoli et al. [13] have followed this strategy to produce mechanically robust PP graphene/GF composites. The authors observed an increase in interfacial interactions between the matrix and GF due to the presence of GNP, while increases of approximately 105% for the modulus and 16% for the tensile strength with the hybrid filler were observed too. Papageorgiou et al. [14] combined both GNPs and GF in order to produce a hybrid PP composite. It was found that the modulus of PP–GNP–GF composites was 3-fold that of the matrix, while the thermal conductivity was increased 5-fold. A competitive effect was also found between GF- and GNP-filled PP, since GFs tend to decrease the thermal stability, while GNPs act towards the increase in the decomposition temperature [15]. 

A further detailed investigation is needed in order to fully evaluate the origins of the interesting phenomenon between GFs and GNPs. On the one hand, an extensive study of thermally stimulated processes such as crystallization and crystallization kinetics is intriguing to control and master the crystallization behavior of PP–GF–GNP composites in order to design materials with desirable properties. On the other hand, the importance of studying the thermal degradation kinetics of PP–GF–GNP composites comes from the need to understand the thermal stability under different conditions because the thermal behavior of plastics can be improved by knowing the parameters of the thermal decomposition process. Many studies on the crystallization kinetics and thermal degradation kinetics of PP have been carried out [17,18,19,20,21]. However, no comprehensive research has been conducted on the isothermal crystallization behavior and thermal degradation kinetics of PP–GF–GNP composites. 

The main goal of this study is to investigate the synergistic effects of GFs and GNPs on the thermal properties of PP composites. For this reason, Differential Scanning Calorimetry was employed to investigate the isothermal crystallization behavior of PP–GF–GNP composites. The kinetic constant and half crystallization time were calculated by the Avrami equation, while the surface free energy of folding was calculated by the Lauritzen–Hoffman theory. Moreover, the effect of each filler along with the filler mixture system on kinetic analysis has also been evaluated by Thermogravimetric Analysis. A detailed study has been performed for the calculation of the effective activation energy using Friedman’s isoconversional differential method. Finally, the kinetic model and the kinetic parameters of the thermal decomposition process were determined by the multivariate non-linear regression method.

## 2. Results and Discussion

### 2.1. Isothermal Crystallization

Understanding the crystallization mechanism is necessary for designing materials with the required properties because the crystallization process influences polymer properties through the crystal structure and morphology established during processing. 

**Figure 1:**
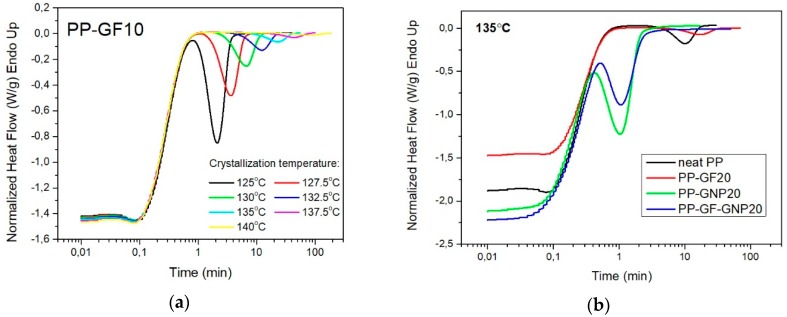
(**a**) Evolution of the exothermic peaks of PP–GF10 composite during the isothermal crystallization at different temperatures; (**b**) a comparative plot of neat PP, PP–GF20, PP–GNP20 and PP–GF–GNP20 composites at 135 °C.

Isothermal crystallization experiments for neat PP and the corresponding composites were performed at different temperatures ranging from 125 to 150 °C. The exothermic curves representing the crystallization of the samples were recorded as a function of time (Figure 1a—only the PP–GF10 sample is presented for brevity reasons), while Figure 1b shows a comparative plot of neat PP, PP–GF20, PP–GNP20 and PP–GF–GNP20 composites at 135 °C. The curves shifted to lower values with a decreasing isothermal crystallization temperature, indicating that the crystallization rate was significantly increased. 

**Figure 2:**
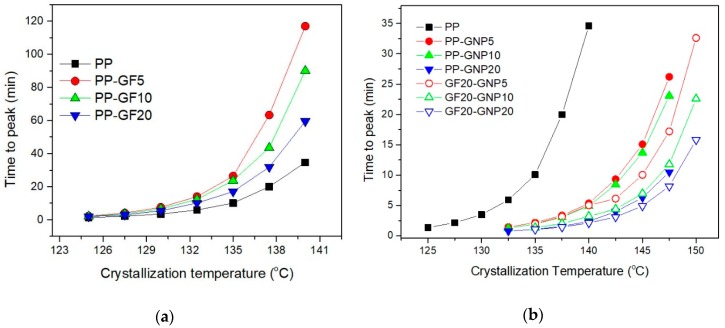
Dependence of the time to peak on the temperature of isothermal crystallization from the melt for (**a**) PP–GF and (**b**) PP–GNP and PP–GF–GNP composites.

The decrease in the time to reach overall crystallization can be used to describe the acceleration of isothermal crystallization. Figure 2 shows the time to the peak for the crystallization exotherm of PP–GF, PP–GNP, and PP–GF–GNP composites. It is obvious that the presence of GNPs accelerated the crystallization (GNPs acted effectively as heterogeneous nucleating agents), while GFs did not enhance the crystallization rates, but on the contrary, retarded the whole crystallization process. Finally, the samples filled with the filler mixture of GF–GNP crystallized at much higher rates due to the presence of GNPs [15]. This anti-nucleation effect caused by GFs is unusual since most inorganic fillers facilitate the heterogeneous nucleation in polymer composites by offering several nucleation sites during the crystallization from the melt. So, a further detailed investigation is needed in order to fully evaluate the origins of the interesting phenomenon between GFs and GNPs.

### 2.2. Avrami Analysis of Isothermal Crystallization

**Figure 3:**
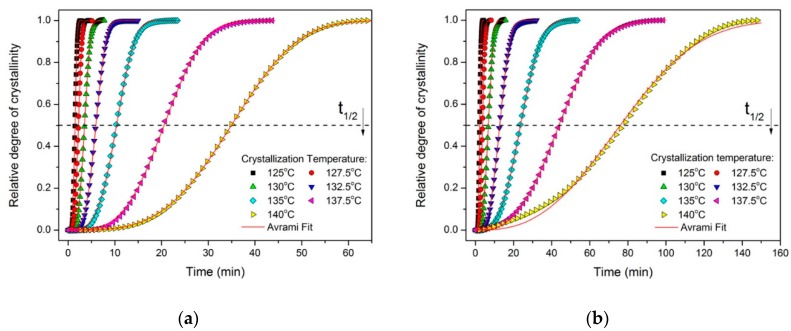
Avrami fit (continuous line) for the data (solid symbols) from the isothermal crystallization of (**a**) neat PP and (**b**) PP–GF10 composite.

The relative degree of crystallinity can be obtained if the assumption that the evolution of crystallinity is linearly proportional to the evolution of heat released during the crystallization phenomenon [22]:

(1) $$X\left(t\right)=\frac{\int_{0}^{t}\left(dH_{c}/dt\right)dt}{\int_{0}^{\infty }\left(dH_{c}/dt\right)dt}$$

where dH_c_ represents the enthalpy of crystallization during an infinitesimal time internal dt, while the limits t and ∞ denote the elapsed time during crystallization and the end of crystallization phenomenon, respectively. Figure 3 shows the relative crystallinity of neat PP and the PP–GF10 composite at different crystallization times in the process of isothermal crystallization. It can be seen that all characteristic sigmoid isotherms shift to the right with increasing isothermal crystallization temperature and the crystallization rate becomes slower.

Then, the isothermal crystallization kinetics of the PP, PP–GF, PP–GNP, and PP–GF-GΝP composites were interpreted in terms of the Avrami equation in order to more precisely analyze the origins of the above-mentioned phenomenon, mainly through nucleation and growth process at a fixed crystallization temperature. The Avrami equation is one of the most used macrokinetic theories of the field and, according to this, the relative degree of crystallinity *X(t)* can be related to the crystallization time according to the expression:

(2) $$X\left(t\right)=1-exp(kt^{n})\;or\;X\left(t\right)=1-exp[-\left(Kt{)}^{n}\right]$$

where n is the Avrami exponent which is related to the nucleation process and *k* is the growth function which is dependent on the nucleation and crystal growth [23,24,25]. The composite Avrami form includes *K* instead of k (where *k = K^n^*) [26]. The non-linear curve fitting procedure based on the Marquardt–Levenberg algorithm was employed for the data from isothermal crystallization since it takes into consideration the whole range of crystallization, compared to the linear fitting process [27]. The simulated theoretical lines can be seen in Figure 3 in comparison with the experimental data and it is obvious that the correlation is very high (R^2^ > 0.99), indicating the efficiency of the Avrami equation for the description of the isothermal crystallization kinetics of the composites. The results from the Avrami analysis are presented in Figure S1 and Table S1. The n values of neat PP were in the vicinity of 2.3–3, similar to most literature reports [28,29], while the Avrami exponent for the GNP-based composites was higher, indicating that there can be an alteration in the growth mode. On the contrary, the GF-based materials presented n values closer to the ones reported in the case of the matrix, but still higher than the matrix. Generally, when n values are close to 2, this is an indication of a two-dimensional growth of the crystals, while when n is close to 3 or higher (as in the case of the composites), this fact is related to heterogeneous nucleation followed by three-dimensional growth. Therefore, all composite materials are exhibiting a 3D growth pattern. Moreover, the increase in the Avrami exponent with increasing *T_c_* is indicative of the sporadic nucleation phenomena which can be observed at higher crystallization temperatures [30]. Regarding the growth rate for the GNP-based composites, the values of *K* are significantly higher than those of neat PP, while once again the materials with the highest loadings exhibit faster rates because of the vast amount of particles and surface provided for heterogeneous nucleation. 

### 2.3. Lauritzen–Hoffman Analysis 

According to the secondary nucleation theory which has been formulated by Hoffman and Lauritzen [31,32], the overall crystallization rate can be controlled by nucleation and transportation of the macromolecules in the melt. The Lauritzen–Hoffman secondary nucleation theory can describe the spherulite growth rate effectively as a function of temperature during isothermal crystallization. Accordingly, G can be expressed by:

(3) $$G=G_{0}exp\left[-\frac{U*}{R\left(T_{c}-T_{\infty }\right)}\right]exp\left[-\frac{K_{g}}{T_{c}\left(\Delta T\right)f}\right]$$

where *G_0_* is the pre-exponential factor, U* and *T_∞_* are the Vogel–Fulcher–Tammann–Hesse (VFTH) parameters describing the transport of the polymer segments across the liquid/crystal interphase, *K_g_* is the nucleation constant and *ΔT* denotes the undercooling. The first exponential term of the above expression is related to the contribution of the diffusion process to the growth rate, while the second exponential term is the contribution of the nucleation process. The generally accepted VFTH parameters are U* = 1500 cal/mol [31,32] and *T_∞_ = (T_g_−30)* K [29,31]. In the present study, the U* parameter and the *T_g_* value of PP was set equal to 1500 cal/mol and 270 K, respectively, [31,33] while the equilibrium melting point was set equal to 485.1 K based on calculations with the non-linear Hoffman–Weeks method [33,34]. Theses substitutions have been widely used in the crystallization study of PP polymer and the corresponding composite systems. It is clear from the above assumptions that the Lauritzen–Hoffman theory provides an approximation and not an absolute value. Thus, the data and calculations presented in this work are mainly for the purpose of qualitative comparison between the PP matrix and its nanocomposites. The calculation of *K_g_* for secondary or heterogeneous nucleation can be obtained by:

(4) $$K_{g}=\frac{jb_{0}\sigma \sigma_{e}T_{m}^{0}}{k_{B}\left(\Delta h_{f}\right)}$$

where j = 4 for regimes I and III and j = 2 for regime II. At a low level of undercooling, crystallization occurs in regime I where the secondary nucleation rate is far less than the surface-spreading rate. At an intermediate level of undercooling, the secondary nucleation rate is comparable to the surface-spreading rate and crystallization takes place in regime II. Regime III emerges at a high level of undercooling where the secondary nucleation rate becomes larger than the surface-spreading rate [31]. The temperature range of crystallization that was selected in this work corresponds to regime III according to Xu et al. [35]. *b_0_* is the thickness of a single stem on the crystal, *σ* is the lateral surface free energy, *Δh_f_* is the enthalpy of fusion and *k_B_* is Boltzmann’s constant. The approximation that the spherulitic growth rate is proportional to the inverse of the halftime of crystallization (*G = 1/t_1/2_*) has been commonly used for this type of studies [33]. Therefore, after the logarithmic transformation of Equation (3), the nucleation constant can be calculated from the expression:

(5) $$ln(G)+\frac{U*}{R\left(T_{c}-T_{\infty }\right)}=ln(G_{0})-\frac{K_{g}}{T_{c}\left(\Delta T\right)f}$$

**Figure 4:**
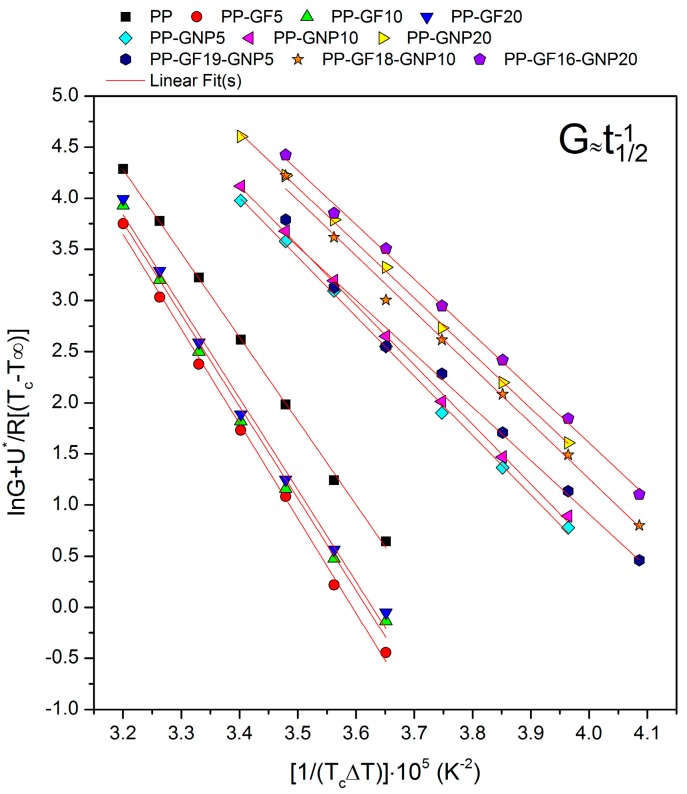
Lauritzen–Hoffman plots of PP and the composites.

Plotting the left-hand side of Equation (5) versus 1*/T_c_(ΔΤ)f* most commonly gives a straight line with a slope and intercept equal to the nucleation constant *–K_g_* and *G_0_*, respectively. The Lauritzen–Hoffman plots for PP and the composites can be seen in Figure 4 and it is obvious that the experimental data are successfully fitted with a linear fitting procedure. 

**Figure 5:**
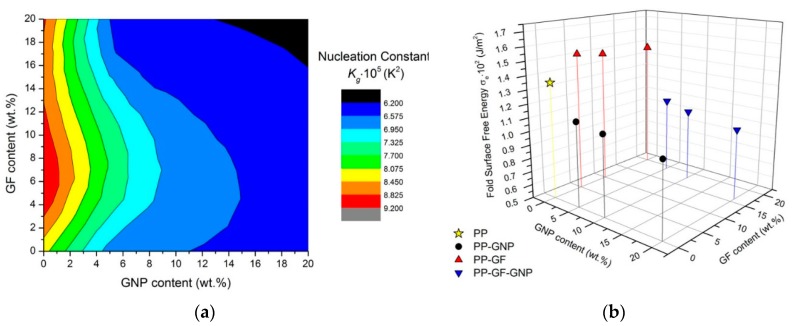
(**a**) Contour plot of the nucleation constant; (**b**) 3D graph of the fold surface free energy for PP and the composites.

The nucleation constant represents the energy that is needed to form a nucleus of a critical size, and it is also related to the lateral and folding surface energy. The results presented in Figure 5 show that the presence of GNPs and their increasing loadings successfully reduces the energy needed for the crystallization of the material. On the contrary, the material filled with GFs demands higher energy values to initiate crystallization and, thus, crystallization is retarded in these samples. The lateral and the fold surface energies are equally important parameters for the crystallization which is governed by secondary nucleation since they are related to both crystal nucleation and growth rates. The lateral surface free energy can be calculated from the empirical equation proposed by Thomas and Staveley [36]:

(6) $$\sigma =\alpha \Delta h_{f}\sqrt{a_{0}b_{0}}$$

where *α* is an empirical constant equal to 0.1 and *α_0_b_0_* represents the cross-sectional area of the polymer chains of PP. According to literature, *α_0_* = 5.46 × 10^−10^ and *b_0_* = 6.26 × 10^−10^ m [37]. Therefore, after obtaining *σ*, the fold surface energy, *σ_e_*, can be calculated by substituting σ into Equation (4). The results for the PP composites are presented in Figure 5b, and it can be seen that the GNP-filled materials present a lower thermodynamic barrier to chain folding and, as a consequence, to polymer crystallization. Once again, GFs seem to increase the energy needed to create a new surface, along with the critical nucleus size needed for crystal growth and make the crystallization phenomenon development more difficult.

### 2.4. Melting Behavior after Isothermal Crystallization

**Figure 6:**
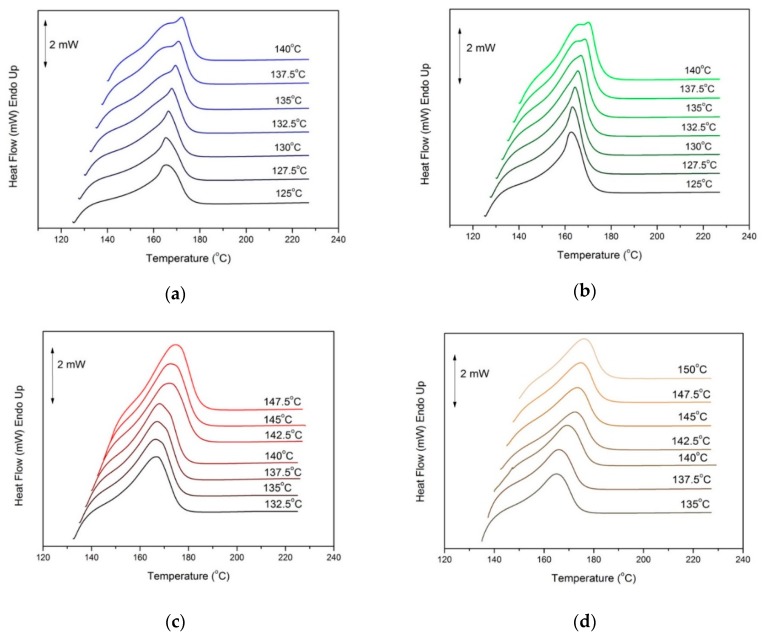
Melting traces of (**a**) PP, (**b**) PP–GF10, (**c**) PP–GNP5, and (**d**) PP–GF18–GNP10 composites after isothermal crystallization at various temperatures.

Neat PP, PP–GF10, PP–GNP5, PP–GFNP18–GNP10 composites were subjected to heating after isothermal crystallization. The heating rate applied was 10 °C/min, and the results can be seen in Figure 6a–d. The melting temperatures remain unchanged, but the melting peaks of neat PP and PP–GF10 composite (Figure 6a,b) present an obvious twinning, especially at higher crystallization temperatures. The specific phenomenon has been described in the past by Hikosaka and Seto [38] and other reports [39,40,41], and it is related to a modification transition mechanism from α_1_ to α_2_ crystals. The monoclinic crystalline structure of PP (α-phase) is the most stable thermodynamically than the other three crystalline phases (β-, γ- and smectic-phase) and presents two variants: the less stable α_1_ phase (phase group C2/c) and the more stable α_2_-phase (P2_1_C) [42]. The generation of α_1_ crystals proceeds during fast cooling from the melt, while α_2_ crystals are formed during the isothermal procedure at higher temperatures. According to Naiki et al. [43], the origin of the differences between the two variants is the methyl group arrangement, which is in perfect order in the α_2_ phase, while it is random in the α_1_ phase. Therefore, the duality of the peak represents the transition from situations with a high degree of disorder, to more ordered situations, which are obtained at isothermal processes from lower to higher crystallization temperatures. 

On the contrary, no twinning can be seen in the GNP-based samples (Figure 6c,d). This can be attributed to the significantly faster crystallization rates of the material, which do not provide enough time in order to generate the more stable α_2_ crystals after the partial melting of α_1_ crystals, even though the crystallization temperatures were much higher than the matrix.

### 2.5. Nucleation Activity

**Figure 7:**
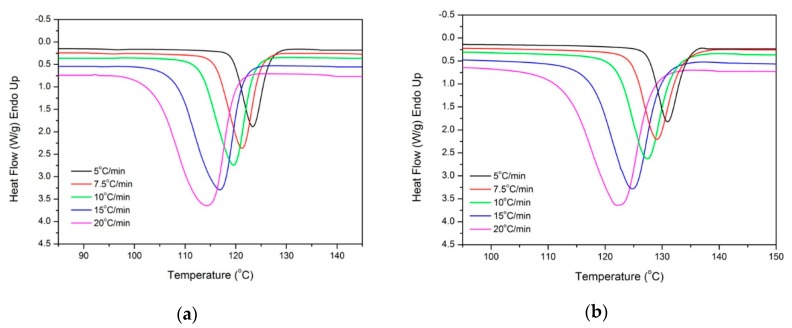
Differential scanning calorimeter (DSC) non-isothermal crystallization exothermic peaks recorded at different cooling rates for (**a**) PP and (**b**) PP–GNP10 composite.

In order to calculate the nucleation activity of the fillers using the method proposed by Dobreva and Gutzow [44], dynamic crystallization experiments were performed for PP and nanocomposite samples at various cooling rates, ranging from 5 to 20 °C/min. The DSC curves were recorded as a function of temperature and they are presented for neat PP and PP–GNP10 composite in Figure 7. It can be seen that the crystallization peak temperature decreased with an increasing cooling rate and that the peaks of the nanocomposite sample can be observed at higher temperatures than neat PP, Figure S2. 

**Figure 8:**
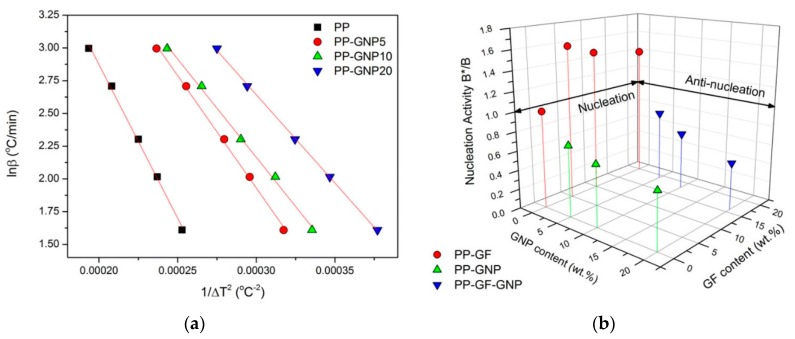
(**a**) Dobreva plots for the evaluation of the nucleation activity of PP-GNP composites. (**b**) 3D plot for the calculated B*/B ratio from Dobreva’s method.

The nucleation activity can be estimated from the ratio φ = B*/B, where B* is a parameter which can be calculated from the following expression:

(7) $$B=\frac{\omega \sigma^{3}V_{m}^{2}}{3nkT_{m}^{0}\Delta S_{m}^{2}}$$

where *ω* is a geometric factor, *σ* is specific energy, *V_m_* is the molar volume of the crystallizing substance, *n* is the Avrami exponent, Δ*S_m_* is the entropy of melting and $T_{m}^{0}$ is the infinite crystal melting temperature. Another way of obtaining *B* is by plotting ln*β* versus the inverse squared degree of supercooling *1/(*Δ*T_p_)^2^* (where Δ*T_p_ = T_m_ − T_p_*) and calculating the slope of these plots:

(8) $$ln\beta =A-\frac{B}{2.303\Delta T_{P}^{2}}\;and\;ln\beta =A-\frac{B^{*}}{2.303\Delta T_{P}^{2}}$$

where *A* is a constant and *B* and B* are the constants related to the homogeneous and heterogeneous nucleation. If the nucleating substance is extremely active, the nucleation activity (B*/B) will be close to zero, while if it is inert, the nucleation activity will be close to unity. Hence, when the nucleation activity values are higher than 1, this fact indicates an anti-nucleation effect of the filler. Plots of ln*β* versus 1/(Δ*T_p_*)^2^ are shown in Figure 8a for the PP–GNP samples, while the results for the nucleation activity are presented in Figure 8b. GNPs in the PP–GNP sample with their large surface area provide increased nucleation activity on the composite samples, which increases with increasing filler content. On the contrary, PP-GFs composites exhibit values of nucleation activity higher than 1, indicating once again their anti-nucleation effect on the PP matrix. Finally, the samples filled with the filler mixture of GF–GNP exhibit a B*/B ratio very close to their PP–GNP counterparts, signifying that the GF in the specific samples are almost inert during the crystallization from the melt and the GNPs are responsible for creating enough surface to initiate crystallization faster at higher temperatures than the matrix. The high nucleation activity of GNP prevailed despite the high GFs content, and the crystallization rates were significantly enhanced under all conditions compared to the matrix. Interestingly, the crystallization rates in the specific set of samples were slightly higher (1–3 °C) than those observed for PP–GNP samples, even though the GFs should not contribute to the specific phenomenon, from previous observations. Most possibly a de-agglomeration occurred due to the increased shear stress between the various particles (loadings ranging from 24–36 wt.%) and a relatively homogeneous distribution of GNPs enabled higher rates of crystallization. Furthermore, the high interconnectivity of GFs [45] may have enabled the formation of a filler network, which is known to act positively towards the increase in the crystallization rates. 

## 3. Kinetic Analysis Based on Thermogravimetric Data

A competitive effect between GFs and GNPs was found for the PP–GF–GNP composite since GFs tend to decrease the thermal stability, while GNPs act towards the increase in the decomposition temperature [15]. A further detailed investigation is needed in order to investigate the reactions which take place during the decomposition process and the mechanisms which describe them. For performing kinetic computations on thermal analysis data, the composites filled with the highest filler content were selected (PP–GF20, PP–GNP20, PP–GF16–GNP20) along with the neat PP. For each sample, four runs were conducted with each one at a different heating rate (5, 10, 15 and 20 °C/min) [46]. 

### 3.1. Isoconversional Methods

**Figure 9:**
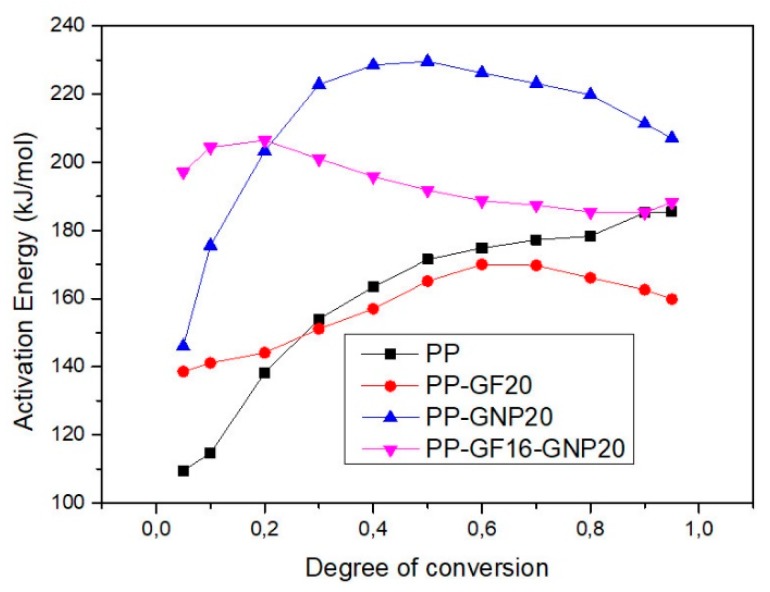
Dependence of the activation energy on the degree of conversion as calculated with Friedman’s method for neat PP, and PP–GF20, PP–GNP20, PP–GF16–GNP20 composites.

The kinetic analysis using data from Thermogravimetric Analysis (TGA) is most commonly performed in two steps: isoconversional and model fitting. Isoconversional methods are generally considered accurate for the processing of thermoanalytical data without any assumption on the reaction mechanism and, apart from the *E-a* dependence, they provide indications on the second step of kinetic analysis, the model-fitting procedure [47,48]. The basis of the isoconversional methods is the assumption that the conversion function *f(α)* does not change with the variation of the heating rate for the whole range of the degree of conversion *a*. In the current work, Friedman’s differential method was used, which was developed by taking the logarithm of the basic rate equation:

(9) $$\frac{\mathrm{da}}{\mathrm{dt}}=k\left(T\right)f\left(a\right)$$

where *T* is the temperature, *t* is the time, and *k(T)* is the rate coefficient which originated from the Arrhenius law $k\left(T\right)=Ae^{-E/RT}$. Therefore, Friedman’s equation takes the form:

(10) $$\;ln\frac{d\alpha }{dt}\;=\;\left[ln{\left(\beta_{i}\frac{d\alpha }{dT}\right)}_{\alpha ,i}\right]\;=\;ln\left[A_{\alpha }f\left(\alpha \right)\right]-\frac{{Ε}_{\alpha }}{RT_{\alpha ,i}}$$

where β = dT/dt. Subscript *i* is the ordinal number of an experiment performed at a given heating rate for non-isothermal conditions. This method is rather accurate because it does not include any mathematical approximations. The activation energy values can be obtained by plotting ln(dα/dT) against 1/T for a constant α value. The results from the application of Friedman’s isoconversional method for the materials filled with the highest filler content, PP–GF20, PP–GNP20, PP–GF16–GNP20 composites can be seen in Figure 9.

From Figure 9, it can be seen that both PP–GNP20 and PP–GF16–GNP20 present higher values of activation energy for the whole range of the degree of conversion. On the contrary, the sample filled with GFs (PP–GF20) presents lower values of E compared to neat PP. Moreover, the samples (PP, PP–GF20, PP–GNP20) present a characteristic pattern on the dependence of *E* on *a.* The curves can be divided into two regions: the first region is extended up to *a* = 0.3–0.4, where the E increases quite rapidly and corresponds to a small mass loss at the beginning of the decomposition. For a >0.4, the *E* values remain almost stable for the rest of the reaction and this region corresponds to the main degradation mechanism. The dependence of E on α suggests that the decomposition is a complex reaction and at least two mechanisms should be considered for the description and fitting of these samples. On the contrary, the fact that the E values for the PP–GF16–GNP20 sample remained almost stable throughout the whole range of α is an indication that the decomposition of this sample may be described with a single-step mechanism. 

### 3.2. Model-Fitting Procedure

**Figure 10:**
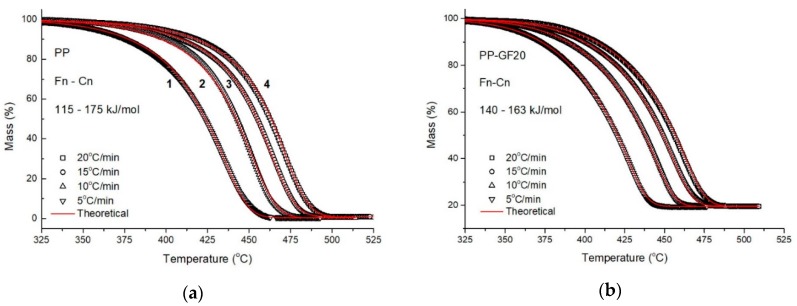

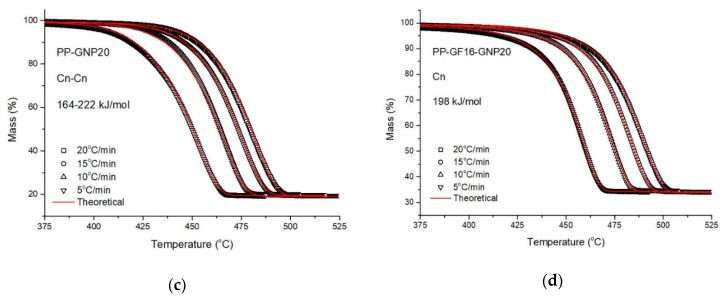
Thermal degradation of (**a**) PP, (**b**) PP–GF20, (**c**) PP–GNP20, and (**d**) PP–GF16–GNP20 composites at different heating rates (**1**) 5 °C/min, (**2**) 10 °C/min, (**3**) 15 °C/min, (**4**) 20 °C/min. The open black symbols represent the experimental data, while the continuous red lines represent the fittings with different models.

The second step of a kinetic analysis based on thermogravimetric data involves the use of fitting methods with mathematical models which can describe the decomposition process. During the procedure, the samples were heated under four different rates (5, 10, 15 and 20 °C/min) and the experimental data were fitted with 16 different kinetic models and their combinations (Figure 10). The kinetic triplet has been estimated for each sample on the basis that the quality of fitting was high enough. The first part of the model-fitting procedure involves the attempt of fitting the experimental data with a single-step model, where the mechanism which is described by the model corresponds to the main mass loss. Therefore, the single-step procedure was applied to all samples under study and only the PP–GF16–GNP20 sample was successfully fitted by a single model, which was the n-th order model with autocatalysis *Cn*:$f\left(a\right)={\left(1-a\right)}^{n}\left(1+K_{cat}\right)X$, where *X* is the reactants and *K_cat_* is a constant. This fact was expected from the isoconversional analysis, since the stable values of the E with increasing α indicated that a simplified procedure could describe the decomposition of the specific sample.

The second part of the procedure involves the testing of different combinations of mathematical models (consecutive or parallel) for the simulation of the experimental data that cannot be fitted with a single-step model. The two mechanisms that describe the decomposition were initially considered consecutive and the combinations that gave the most accurate fitting results were *Fn-Cn* for the neat PP and the PP–GF20 sample, where *Fn* is an n-th order model: $f\left(a\right)={\left(1-a\right)}^{n}$, while the PP–GNP20 sample exhibited a different mechanism (*Cn-Cn*). Once again, the use of the two mechanisms was expected from the *E-a* dependency that was observed in the isoconversional methods (Figure 9). The results from the model fitting procedure are summarized in Table 1. It is worthwhile noting that the degradation of neat PP was found to take place into two stages in agreement with the literature; the first stage, the initial small mass loss, was simulated with an n-th order model, while the second stage was attributed to the main degradation mechanism and was simulated with an n-th order model with autocatalysis [21].

From the results presented in Table 1, it can be seen that the correlation quality of all fittings (neat PP, PP–GF20, PP–GNP20 and PP–GF16–GNP20 composites) was very high for all combinations. Besides the PP–GF16–GNP20 composite, which exhibited a single value of activation energy and a different decomposition pattern, an increase in the activation energy values was recorded for all samples during the transition from the 1st to the 2nd step. However, the PP–GF20 sample presented lower E than the matrix at the 2nd step and thus affected the thermal stability of the matrix. Moreover, the neat PP and PP–GF20 samples were fitted with the same models (*Fn-Cn*), which indicates that GFs did not alter the decomposition mechanism. On the contrary, GNPs altered the 1st step of the decomposition for the PP–GNP20 sample (*Cn-Fn*), while they totally changed the decomposition of PP–GF16–GNP20. The effect of GNP filler is more pronounced, increasing the activation energy of thermal degradation in agreement with the calculated dependence of E on α (Figure 9) and the significant thermal stability enhancement of the PP matrix [15]. This increase in the activation energy of the nanocomposites is associated with the two-dimensional planar structure of GNPs, and it is compatible with the nanoconfinement concept as described by Chen et al., according to which, the presence of GNPs creates areas where the macromolecular chains of the matrix are confined, disturbing their regular coil conformation [15,49,50,51]. Thus, the mobility of polymer matrix is restricted, and the chemical reactivity of the corresponding chains is lower, increasing the activation energy and eventually further retarding the thermal degradation of the nanocomposites. The nanoconfinement concept can also explain the high activation energy of the PP–GF16–GNP20 composite since the simultaneous presence of GFs and GNPs occupies an extensive area in the volume of the composite, forming a well-distributed filler network and further confining the movement of the PP macromolecules. It should be noted that the density of GNPs is significantly lower than that of GFs and the nanofiller specific surface area is almost two orders of magnitude greater than that of GFs and, thus, GNP surface area dominates.

## 4. Materials and Methods

### 4.1. Materials

PP homopolymer was provided by Lyondellbasell with the commercial name Moplen HP501L and exhibited a flow index of 6 g/10min and melt density of 900 kg/m^3^. The glass-filled PP was provided by ALBIS under the commercial name Altech PP-H A 2020/159 GF20 CP, which is a PP homopolymer product filled with 20 wt.% GF, and it exhibited a melt density of 1040 kg/m^3^. The exfoliated GNPs (xGNP-25) were produced by sulfuric-based intercalated graphite and were obtained from XG Sciences (East Lansing, MI, USA). The nanoplatelets exhibited a mean platelet diameter of 25 μm and an average thickness of 6–8 nm. Their oxygen content was less than 1%, while the residual acid content was less than 0.5 wt.%.

The extrusion process was performed with a twin-screw extruder (Thermo Scientific HAAKE MiniLab micro compounder, Karlsruhe, Germany) at 190 °C and 100 rpm, while the mixing took place for 12 min. Afterwards, the prepared materials were hot pressed in order to prepare films of different thicknesses appropriate for each type of following measurements. The GNP-filled materials were named PP–GNPx throughout the manuscript, where x is the filler content at wt.% (x = 5, 10, 20 wt.%). The same applies to the samples filled with GF (PP–GFx). To produce the PP–GF–GNP composite, the material filled with 20 wt.% GF (PP–GF20) was used as a matrix and the GNPs were added in the melt mixing process. This caused a dilution of the GF content in the final batch and, at increasing GNP content, lowered the amount of GFs. So, three sets of samples were prepared, namely PP–GF19–GNP5, PP–GF18–GNP10, and PP–GF16–GNP20.

### 4.2. Differential Scanning Calorimetry (DSC)

A TA Q100 differential scanning calorimeter (DSC) from TA Instruments (New Castle, UK), calibrated with indium and zinc standards, was used for the study of crystallization and melting of the composites. Dry nitrogen gas was purged into the DSC cell with a flow rate of 50 mL/min. The weight of the samples was 5 ± 0.2 mg, while they were sealed in aluminium pans and heated at a heating rate 20 °C/min to 240 °C, which is above the equilibrium melting point of PP, for 5 min. The specific procedure was followed in order to erase the thermal history of the samples. For the isothermal crystallization study, the experiments were carried out according to the detailed procedure recommended by Lorenzo et al. [50]. Briefly, the samples were rapidly cooled from 240 °C to the crystallization temperature at a rate of 80 °C/min and then held at the specific temperature for a time period. The crystallization exothermic peak was then recorded. Heating scans were performed at a rate of 10 °C/min. It should be noted that the selected isothermal crystallization temperatures of the GNP-containing samples were not the same for every set of materials because the instrument was not able to equilibrate before reaching the relatively low crystallization temperatures. This happened since the samples crystallized very fast and the apparatus could not settle properly at the specific temperature. For this reason, in order to obtain a well-defined crystallization exotherm and for the avoidance of errors from baseline determination and onset time, higher temperatures were selected to allow for more time for the DSC to stabilize and record the curve properly. The measurement procedure was performed according to the detailed guidelines from Lorenzo et al. [52] for the isothermal crystallization kinetics measurements and the proper use of the Avrami equation to fit the data. For the non-isothermal crystallization, cooling scans were performed at rates of 5, 7.5, 10, 15 and 20 °C/min. A fresh sample was used for each run.

### 4.3. Thermogravimetric Analysis (TGA)

Thermogravimetric analysis experiments were carried out using a SETARAM SETSYS TG-DTA 16/18 instrument (Caluire, France). Samples of 5 ± 0.5 mg were placed in alumina crucibles, while an empty alumina crucible was used as a reference. According to the recommendations of ICTAC [45] for the kinetic analysis, the matrix and the composite materials filled with the highest filler content (PP–GF20, PP–GNP20, PP–GF16–GNP20) were heated from room temperature up to 600 °C in a 50 mL/min flow of N_2_ at four different rates, namely 5, 10, 15 and 20 °C/min.

## 5. Conclusions

GNPs and GFs were inserted individually and simultaneously in an isotactic PP matrix; the nucleation, crystallization behavior, and thermal degradation kinetics were studied under a wide variety of conditions. For the samples containing GNPs, the high number of heterogeneous nucleating surfaces along with the geometrical characteristics of the filler enabled the composite samples to crystallize at higher rates in isothermal conditions. On the contrary, the presence of GFs retarded the crystallization procedure. Crystallization at higher temperatures enabled crystalline perfection in the matrix and observation of the melting of both imperfect and perfect crystals of PP and PP–GF samples, while for the PP–GNP and PP–GF–GNP composites, the faster crystallization rates did not allow enough time for the crystals to develop a perfect crystalline structure. The anti-nucleation activity of the GF was also verified from Dobreva’s method, and it was attributed to the extensive formation of a rigid amorphous fraction in the material with restricted chain mobility, which did not enhance the crystallization rates. Isoconversional and model fitting methods were successfully employed for the study of the decomposition of PP composites, and the results showed that the presence of GNPs altered the decomposition mechanism of the composites. In detail, the Cn model best described the thermogravimetric data for the PP–GF16–GNP20 composite, while the Fn-Cn models and Cn-Cn models better described the two decomposition stages of the PP–GF20 and PP–GNP20 composites, respectively. The effect of GNPs is more pronounced compared to GFs, increasing the activation energy due to the two-dimensional planar structure of GNPs and the mobility restriction of the segmental movement of the PP matrix. The presence of GNPs creates areas where the macromolecular chains of the matrix are confined, disturbing their regular coil conformation and restricting their movement. In conclusion, the easily produced GNP-based PP composites can be used in a number of advanced applications where good thermal and mechanical performance is needed.

## Figures and Tables

**Table 1:** Calculated values of activation energy (E), pre-exponential factor (A), reaction order (n), branching rate constant (K_cat_) and correlation coefficient (R^2^) for PP and the composites.

	**1st Step**					
**Sample**	**Mechanism**	**E (kJ/mol)**	**logA (s^−1^)**	**N**	**logK_cat_**	**R^2^**
PP	*Fn*	115	6.3	0.4	-	0.9998
PP–GF20	*Fn*	140	8.5	0.6	-	0.9998
PP–GNP20	*Cn*	163	9.2	0.6	0.6	0.9999
PP–GF16–GNP20	*Cn*	198	11.4	0.7	0.7	0.9999
	**2nd Step**					
**Sample**	**Mechanism**	**E (kJ/mol)**	**LogA**	**N**	**LogK_cat_**	**R^2^**
PP	*Cn*	175	10.3	1.0	0.3	0.9998
PP–GF20	*Cn*	163	9.4	0.9	0.6	0.9998
PP–GNP20	*Cn*	222	13.5	0.8	0.2	0.9999

## Supplementary Materials

The supplementary materials are available online, Table S1: Avrami parameters of PP and the composite samples at different isothermal crystallization temperatures, Figure S1: (a) Avrami exponent of PP and nanocomposites. The n values of the matrix have been plotted using both symbol and line in order to separate them easier from the values of the composites; (b) growth function *K* of all materials under study, Figure S2.: Evolution of the crystallization peak temperature as a function of cooling rate for PP and nanocomposites.
