# Supplementary material for: Kinetics of Crystallization and Thermal Degradation of an Isotactic Polypropylene Matrix Reinforced with Graphene/Glass-Fiber Filler
Source: Molecules. 2019 May 23;24(10):1984. doi: 10.3390/molecules24101984 (PMC6572227; doi:10.3390/molecules24101984)
Supplement: Supplementary file 1 [file molecules-24-01984-s001.pdf]

molecules

MDPI

Article

Kinetics of Crystallization and Thermal Degradation of an Isotactic Polypropylene Matrix Reinforced with Graphene/Glass-Fiber Filler

Evangelia Tarani [1], George Z. Papageorgiou [2], Dimitrios N. Bikiaris [3], and Konstantinos Chrissafis [1,*]

Supplementary Materials

Table 1. Avrami parameters of PP and composite samples at different isothermal crystallization temperatures.

	PP			PP-GF5			PP-GF10			PP-GF20		
Crystallization Temperature (°C)	n	K (min^{-n})	$t_{1/2}$ (min)	n	K (min^{-n})	$t_{1/2}$ (min)	n	K (min^{-n})	$t_{1/2}$ (min)	n	K (min^{-n})	$t_{1/2}$ (min)
125	2.3	0.62	1.3	3.2	0.39	2.3	3.1	0.41	1.92	3.2	0.4	1.8
127.5	2.4	0.41	2	3.2	0.22	4.4	3.2	0.24	3.7	3.4	0.21	3.4
130	2.5	0.25	3.4	3.3	0.11	7.9	3.2	0.13	7	3.5	0.11	6.4
132.5	2.9	0.15	5.8	3.4	0.06	14.2	3.4	0.07	13	3.4	0.07	12.1
135	2.8	0.08	10.3	3.5	0.02	25.4	3.4	0.03	23.6	3.6	0.04	21.6
137.5	2.9	0.04	20.4	3.4	0.01	56.8	3.6	0.02	43.9	3.6	0.03	40.3
140	3	0.01	35	3.6	0.001	104	3.5	0.01	76.2	3.7	0.02	70.1
	PP-GNP 5			PP-GNP10			PP-GNP20					
Crystallization Temperature (°C)	n	K (min^{-n})	$t_{1/2}$ (min)	n	K (min^{-n})	$t_{1/2}$ (min)	n	K (min^{-n})	$t_{1/2}$ (min)			
132.5	3.1	0.6	1.5	3.5	0.7	1.3	3.7	1.1	0.8			
135	3.4	0.42	2.1	3.6	0.47	1.9	3.8	0.8	1.1			
137.5	3.5	0.27	3.2	3.9	0.3	2.9	4	0.55	1.6			
140	3.7	0.15	5.2	4	0.21	4.7	4	0.32	2.4			
142.5	4	0.09	9.4	4.1	0.16	8.4	3.9	0.24	4.1			
145	4.1	0.04	15.2	4.1	0.1	13.7	4.1	0.17	6.6			
147.5	4.2	0.01	25.9	4.4	0.013	23.1	4.5	0.1	11.3			
	PP-GF19-GNP5			PP-GF18-GNP10			PP-GF16-GNP20					

Crystallization Temperature (°C)	n	K (min^{-n})	$t_{1/2}$ (min)	n	K (min^{-n})	$t_{1/2}$ (min)	n	K (min^{-n})	$t_{1/2}$ (min)	
135	2.7	0.45	1.7	2.8	0.63	1.1	2.6	0.83	0.9	
137.5	2.8	0.27	3.1	3	0.42	1.9	2.9	0.59	1.5	
140	2.9	0.13	5.2	3.1	0.26	3.3	3	0.41	2	
142.5	2.9	0.08	6.4	3.1	0.18	4.6	3.4	0.26	3.3	
145	3	0.06	10.8	3.3	0.11	7.4	4	0.16	5.3	
147.5	3.2	0.04	18.1	3.8	0.07	12.7	4.2	0.09	8.9	
150	3.8	0.02	33.7	4.1	0.06	24	4.3	0.04	17.7	

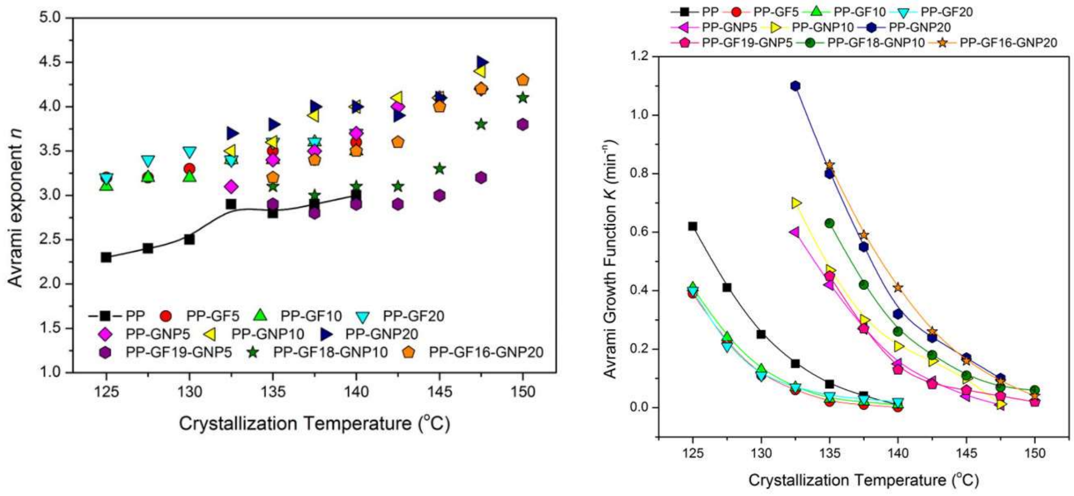

(**b**)

(**a**)

Figure 1. (**a**) Avrami exponent of PP and nanocomposites. The n values of the matrix have been plotted using both symbol and line in order to separate them easier from the values of the composites; (**b**) growth function *K* of all materials under study.

The variation of the crystallization temperature versus the cooling rate, for each sample, can be seen in Figure S2. For the PP-GNP samples, the presence of the "foreign" substances at high loadings along with their wide surface area, provide a lot of sites enabling the heterogeneous nucleation at temperatures significantly higher than that of the neat PP, reaching even 12°C for the material with the highest loading. Interestingly and similarly to the observations from the isothermal crystallization, GF didn't facilitate crystallization but retarded the overall phenomenon since the crystallization peaks were observed at temperatures higher than the matrix. The sample filled with 5 wt.% GF presented the highest retardation of the phenomenon, but the temperature differences were quite small for the specific set of samples. Finally, the presence of GNP in the PP-GF-GNP samples attributed high crystallization rates, and the GF didn't seem to affect significantly the crystallization, based on the peak temperatures.

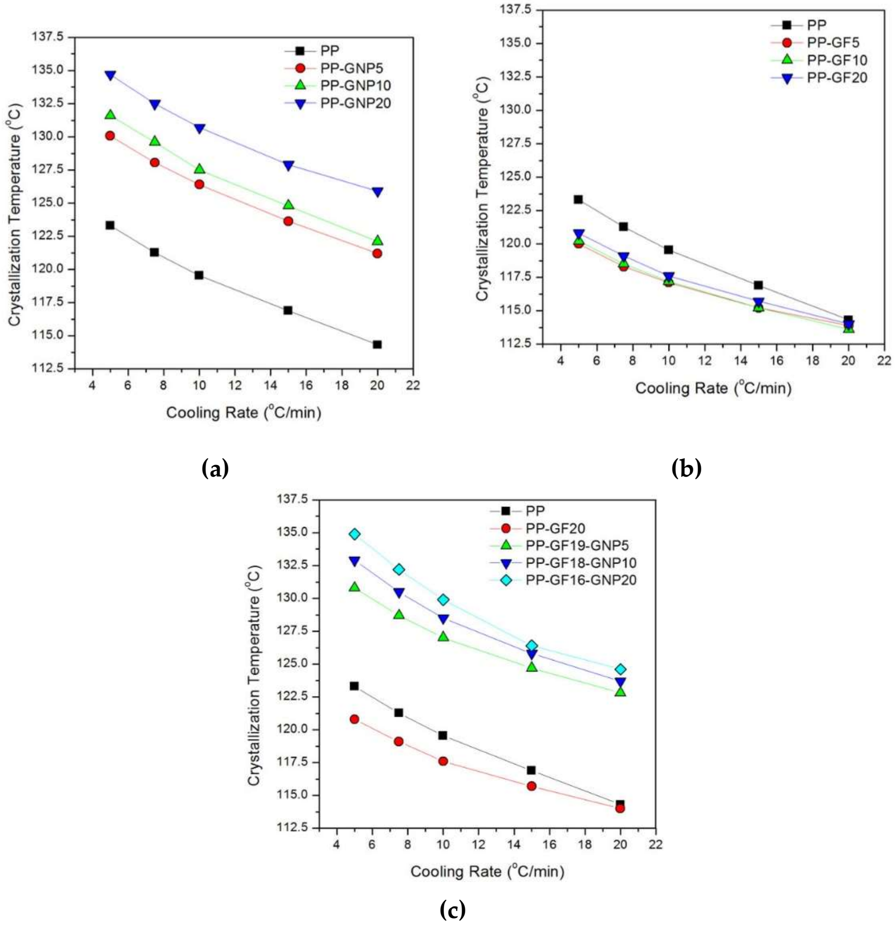

Figure 2. Evolution of the crystallization peak temperature as a function of cooling rate for PP and nanocomposites.
